# Integer time series models for tuberculosis in Africa

**DOI:** 10.1038/s41598-023-38707-4

**Published:** 2023-07-15

**Authors:** Oluwadare O. Ojo, Saralees Nadarajah, Malick Kebe

**Affiliations:** 1 Department of Statistics, Federal University of Technology, Akure, Nigeria; 2 Department of Mathematics, University of Manchester, Manchester, M13 9PL UK; 3 Department of Mathematics, Howard University, Washington, DC 20059 USA

**Keywords:** Infectious diseases, Software, Statistics

## Abstract

Tuberculosis, an airborne disease, is the deadliest human infectious disease caused by one single agent. The African region is among the most affected and most burdensome area in terms of tuberculosis cases. In this paper, we modeled the number of new cases of tuberculosis for 2000–2021 by integer time series. For each African country, we fitted twenty different models and selected the model that best fitted the data. The twenty models were mostly based on the number of new cases following either the Poisson or negative binomial distribution with the rate parameter allowed to vary linearly or quadratically with respect to year. The best fitted models were used to give predictions for 2022–2031.

## Introduction

Tuberculosis (TB), an airborne disease, is the deadliest human infectious disease caused by one single agent, *Mycobacterium tuberculosis* (Mtb). The bacteria usually attacks the lungs, but it can also attack any part of the body such as the kidney, spine, and brain (CDC^1^). Fortunately, not everyone infected with TB bacteria becomes sick. Mtb is believed to have originated in East Africa around three million years ago but the first known description of TB was found in India and China as early as 3300 and 2300 years ago, respectively. While TB was a major cause of death in the 19th and early 20th centuries, the development of effective antibiotics in the mid-20th century led to a dramatic decline in the incidence of the malady (Dutta^2^).

TB continues to kill more than a million deaths per year in spite of a 47% drop in TB mortality rate since 1990. In 2021, 10.6 million people worldwide were diagnosed with TB with 1.6 million deaths, including 187,000 people with HIV, a major driver of TB (World Health Organization^3^).

Chakaya et al.^4^ believed that the COVID-19 pandemic worsened an already sub-optimal global TB response. According to the World Health Organization^5^, the disruption and reallocation of resources due to the COVID-19 pandemic has reversed years of global progress in reducing the number of people who die from TB with the estimated number of deaths in 2020 back to the level of 2017 leading to a reduction of only 9.2% instead of the targeted 35% reduction in the number of TB deaths between 2015 and 2020.

While TB can affect anyone irrespective of creed, the highest burden is in adult men, and those living in Africa and Asia. In this paper, we focus on African incidences. There are a number of reasons why TB is common in Africa. These include (i) poverty-which causes limited access to health care; (ii) HIV/AIDS-which weakens the immune system, making people more susceptible to TB. In Africa, about 25% of people living with HIV also have TB; (iii) weak health systems causing difficulties to diagnose and treat TB; (iv) a lack of awareness-which can lead to people not seeking treatment, inevitably causing the disease spread.

**Figure 1:**
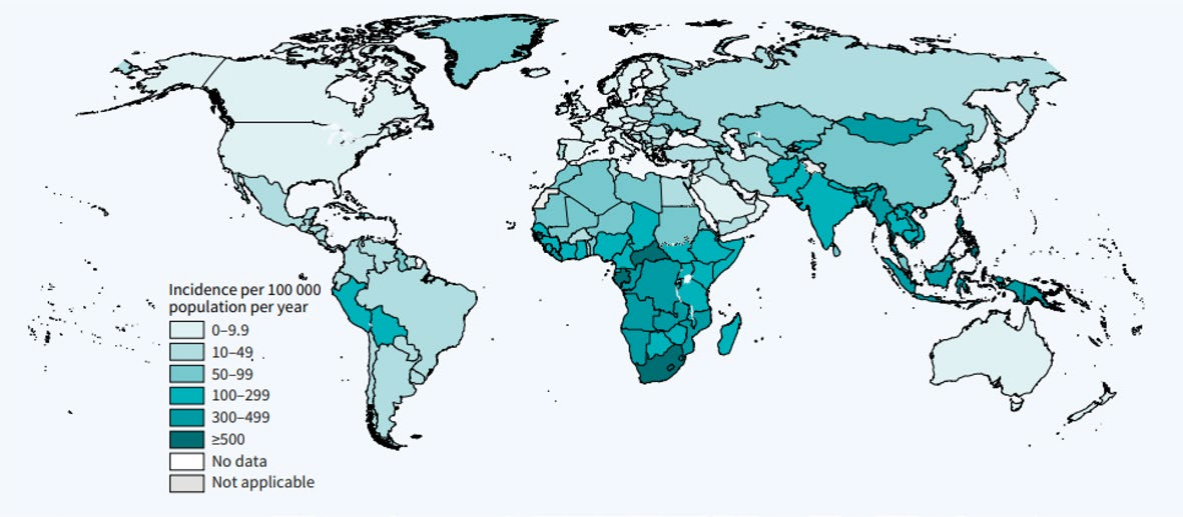
Estimated TB incidence rates, 2021. *Source* World Health Organization^3^ Global Tuberculosis Report.

Figure 1 shows the estimated TB incidence rates in the world. Most of the countries having high TB incidence rates appear to be in Africa.

According to Silva et al.^6^, fulfilling the UN Sustainable Development Goal (SDG) of ending the TB epidemic by 2030 is appearing more and more improbable. Silva et al.^6^ conducted a study estimating the economic impact of not meeting the target until 2045, including those due to excess deaths resulting from COVID-19-related disruptions to TB services for the period 2020–2050. They found that if the current rate of TB deaths continues, 31.8 million people will die from TB between 2020 and 2050, leading to an economic loss of $17.5 trillion. Meeting a 90 percent reduction in TB deaths by 2030 would save 23.8 million lives and $13.1 trillion. This would save $3.0 trillion in additional economic losses and likely spare the lives of 5.7 million additional individuals as compared to if the 90% reduction was to be met in 2045 instead. In short, meeting the SDG TB mortality target is essential to saving lives and preventing economic losses.

One way to model TB is through mathematical epidemiological models (see, for example, Ozcaglar et al.^7^). These models have the advantage of taking into account the dynamics of the disease and the researcher’s understanding of the biological principles that drive the spread of the malady. For example, accounting for the slow spread of TB may result in more precise predictions for the long term. We are not so much interested in the dynamics of the disease as interest here is in the count of the number of people infected with the disease. Therefore, we use more of a statistical method.

Our modeling approach does not involve simulations, but instead fits a curve to the historical data. This way of modeling simply reflects the observed relationship between variables and attempts to forecast future results based on the past data (Dhlakama et al.^8^).

There has been research concerning the modeling of TB. Liu et al.^9^ developed a TB model incorporating seasonality based on data from China. They developed a compartmental model to describe TB seasonal incidence rate by incorporating periodic coefficients and discovered that there is a seasonal pattern of new TB cases, with those numbers peaking in late spring to early summer. Their research attributed the seasonal pattern to the Chinese Spring Festival, and/or to the more frequent viral infections like flu, which can lead to reactivation of the Mtb.

Using 462,214 pulmonary TB cases over a period of 10 years as training data, Liu et al.^10^ used two different approaches to predict the number of TB cases in Jiangsu Province, China. The first approach, autoregressive integrated moving average (ARIMA), is a statistical method that uses past data to predict future trends. The second approach, back-propagation neural network (BPNN), is a neural network method that uses statistical machine learning to learn from data. Both approaches were able to predict seasonality and trend of pulmonary TB in the Chinese population, but the BPNN approach was slightly more accurate.

Using multilevel modelling, Dhlakama et al.^8^ investigated factors that influence self-reported TB cases from 2008 to 2017. They discovered several variables that had a significant impact on TB such as marital status, gender, race, unemployment, other diseases, exercise patterns, smoking patterns, health consultation, asthma diagnosis, diabetes diagnosis, housing quality and household. The results were the same using both frequentist and Bayesian models even with informative priors.

In this paper, we model the prevalence of TB in Africa as calculated by incidence rate per 100,000 people. We model the distribution of the number of new cases of TB in a given year conditioned on the history of the number of cases up to that year. For each country, we fitted the incidence data using 20 models for integer time series and selected the best fitting model. Most of the twenty models were based on the number of new cases following either the Poisson or negative binomial distribution with the rate parameter allowed to vary linearly or quadratically with respect to year. Others were based on the number of new cases following either the Poisson or negative binomial distribution with the rate parameter allowed to depend on previous number of new cases. There are no papers modeling incidence rates of TB for all African countries.

The rest of the paper is structured as follows. “Data” section discusses the data, “Models” section discusses the models used to fit the data, “Results and discussion discusses the results and the paper is concluded in “Conclusion” section.

## Data

According to the World Bank, TB incidence is the rate per 100,000 population of new and relapse TB cases that occur in a year. This number covers all types of TB such as other members of the Mtb complex and also cases in people living with HIV (World Bank^11^).

**Figure 2:**
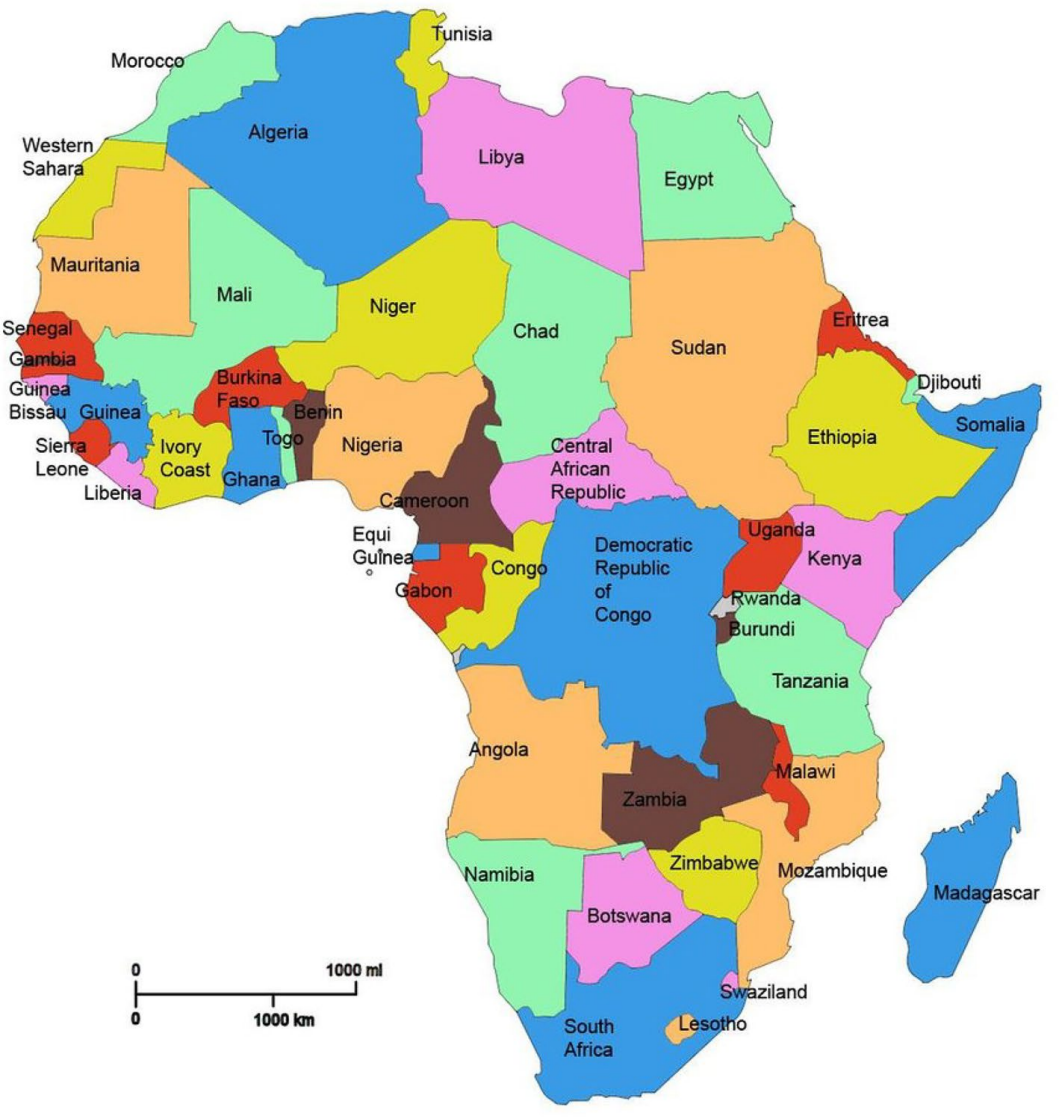
The countries in Africa.

The data are new cases of TB reported in $2000, 2001, \ldots , 2021$ for the following African countries: Algeria, Angola, Benin, Botswana, Burkina Faso, Burundi, Cabo Verde, Cameroon, Central African Republic, Chad, Comoros, the Democratic Republic of Congo, Republic of Congo, Cote d’Ivoire, Djibouti, Egypt, Equatorial Guinea, Eritrea, Eswatini, Ethiopia, Gabon, the Gambia, Ghana, Guinea, Guinea-Bissau, Kenya, Lesotho, Liberia, Madagascar, Malawi, Mali, Mauritania, Mauritius, Morocco, Mozambique, Namibia, Niger, Nigeria, Rwanda, Sao Tome and Principe, Senegal, Seychelles, Sierra Leone, Somalia, South Africa, Sudan, Tanzania, Togo, Tunisia, Uganda, Zambia and Zimbabwe, see Fig. 2. The data were obtained from https://data.worldbank.org/indicator/SH.TBS.INCD. We were not able to obtain the data for Libya, South Sudan or Western Sahara.

## Models

Let $Z_t$ denote the number of new cases of TB reported in year *t*, $t = 2000, 2001, \ldots , 2021$. Let $\mathcal{F}_t$ denote the history of the number of new cases up to and including year *t*. For each of the fifty two countries, the following models were fitted

- $Z_t \mid \mathcal{F}_{t - 1} \sim$ Poisson $\left( \beta _0 \right)$, referred to as the identity Poisson model;
- $Z_t \mid \mathcal{F}_{t - 1} \sim$ Poisson $\left( \exp \left( \beta _0 \right) \right)$, referred to as the log Poisson model;
- $Z_t \mid \mathcal{F}_{t - 1} \sim$ Negative Binomial $\left( \beta _0, \phi \right)$, referred to as the identity negative binomial model;
- $Z_t \mid \mathcal{F}_{t - 1} \sim$ Negative Binomial $\left( \exp \left( \beta _0 \right) , \phi \right)$, referred to as the log negative binomial model;
- $Z_t \mid \mathcal{F}_{t - 2} \sim$ Poisson $\left( \beta _0 + \beta _1 Z_{t - 1} \right)$, referred to as the identity Poisson model regressed on the previous observation;
- $Z_t \mid \mathcal{F}_{t - 2} \sim$ Poisson $\left( \exp \left( \beta _0 + \beta _1 Z_{t - 1} \right) \right)$, referred to as the log Poisson model regressed on the previous observation;
- $Z_t \mid \mathcal{F}_{t - 2} \sim$ Negative Binomial $\left( \beta _0 + \beta _1 Z_{t - 1}, \phi \right)$, referred to as the identity negative binomial model regressed on the previous observation;
- $Z_t \mid \mathcal{F}_{t - 2} \sim$ Negative Binomial $\left( \exp \left( \beta _0 + \beta _1 Z_{t - 1} \right) , \phi \right)$, referred to as the log negative binomial model regressed on the previous observation;
- $Z_t \mid \mathcal{F}_{t - 3} \sim$ Poisson $\left( \beta _0 + \beta _1 Z_{t - 1} + \beta _2 Z_{t - 2} \right)$, referred to as the identity Poisson model regressed on the two previous observations;
- $Z_t \mid \mathcal{F}_{t - 3} \sim$ Poisson $\left( \exp \left( \beta _0 + \beta _1 Z_{t - 1} + \beta _2 Z_{t - 2} \right) \right)$, referred to as the log Poisson model regressed on the two previous observations;
- $Z_t \mid \mathcal{F}_{t - 3} \sim$ Negative Binomial $\left( \beta _0 + \beta _1 Z_{t - 1} + \beta _2 Z_{t - 2}, \phi \right)$, referred to as the identity negative binomial model regressed on the two previous observations;
- $Z_t \mid \mathcal{F}_{t - 3} \sim$ Negative Binomial $\left( \exp \left( \beta _0 + \beta _1 Z_{t - 1} + \beta _2 Z_{t - 2} \right) , \phi \right)$, referred to as the log negative binomial model regressed on the two previous observations;
- $Z_t \mid \mathcal{F}_{t - 1} \sim$ Poisson $\left( \beta _0 + \beta _1 t \right)$, referred to as the identity Poisson model regressed linearly with respect to year;
- $Z_t \mid \mathcal{F}_{t - 1} \sim$ Poisson $\left( \exp \left( \beta _0 + \beta _1 t \right) \right)$, referred to as the log Poisson model regressed linearly with respect to year;
- $Z_t \mid \mathcal{F}_{t - 1} \sim$ Negative Binomial $\left( \beta _0 + \beta _1 t, \phi \right)$, referred to as the identity negative binomial model regressed linearly with respect to year;
- $Z_t \mid \mathcal{F}_{t - 1} \sim$ Negative Binomial $\left( \exp \left( \beta _0 + \beta _1 t \right) , \phi \right)$, referred to as the log negative binomial model regressed linearly with respect to year;
- $Z_t \mid \mathcal{F}_{t - 1} \sim$ Poisson $\left( \beta _0 + \beta _1 t + \beta _2 t^2 \right)$, referred to as the identity Poisson model regressed quadratically with respect to year;
- $Z_t \mid \mathcal{F}_{t - 1} \sim$ Poisson $\left( \exp \left( \beta _0 + \beta _1 t + \beta _2 t^2 \right) \right)$, referred to as the log Poisson model regressed quadratically with respect to year;
- $Z_t \mid \mathcal{F}_{t - 1} \sim$ Negative Binomial $\left( \beta _0 + \beta _1 t + \beta _2 t^2, \phi \right)$, referred to as the identity negative binomial model regressed quadratically with respect to year;
- $Z_t \mid \mathcal{F}_{t - 1} \sim$ Negative Binomial $\left( \exp \left( \beta _0 + \beta _1 t + \beta _2 t^2 \right) , \phi \right)$, referred to as the log negative binomial model regressed quadratically with respect to year,

where $\beta _0$, $\beta _1$ and $\beta _2$ are the regression coefficients. These models are due to Fokianos et al.^12^, Fokianos and Tjostheim^13^, Fokianos and Fried^14^ and Christou and Fokianos^15,16^. The models due to Christou and Fokianos^15^ are based on the negative binomial distribution. The models due to the others are based on the Poisson distribution. Both Poisson and negative binomial distributions are commonly implemented when dealing with count data.

The stated models were fitted by the method of maximum likelihood. That is, by maximizing

$$\begin{aligned}{} & {} \displaystyle L_1 \left( \beta _0 \right) = \prod _{t = 2000}^{2021} \frac{\beta _0^{Z_t} \exp \left( -\beta _0 \right) }{Z_t!},\\{} & {} \displaystyle L_2 \left( \beta _0 \right) = \prod _{t = 2000}^{2021} \frac{\exp \left( Z_t \beta _0 \right) \exp \left[ -\exp \left( \beta _0 \right) \right] }{Z_t!},\\{} & {} \displaystyle L_3 \left( \beta _0, \phi \right) = \prod _{t = 2000}^{2021} \left[ {Z_t + \beta _0 - 1 \atopwithdelims ()Z_t} (1 - \phi )^{\beta _0} \phi ^{Z_t} \right] ,\\{} & {} \displaystyle L_4 \left( \beta _0, \phi \right) = \prod _{t = 2000}^{2021} \left[ {Z_t + \exp \left( \beta _0 \right) - 1 \atopwithdelims ()Z_t} (1 - \phi )^{\exp \left( \beta _0 \right) } \phi ^{Z_t} \right] ,\\{} & {} \displaystyle L_5 \left( \beta _0, \beta _1 \right) = \prod _{t = 2000}^{2021} \frac{\left( \beta _0 + \beta _1 Z_{t - 1}\right) ^{Z_t} \exp \left( -\beta _0 - \beta _1 Z_{t - 1} \right) }{Z_t!},\\{} & {} \displaystyle L_6 \left( \beta _0, \beta _1 \right) = \prod _{t = 2000}^{2021} \frac{\exp \left[ Z_t \left( \beta _0 + \beta _1 Z_{t - 1}\right) \right] \exp \left[ -\exp \left( \beta _0 + \beta _1 Z_{t - 1}\right) \right] }{Z_t!},\\{} & {} \displaystyle L_7 \left( \beta _0, \beta _1, \phi \right) = \prod _{t = 2000}^{2021} \left[ {Z_t + \beta _0 + \beta _1 Z_{t - 1} - 1 \atopwithdelims ()Z_t} (1 - \phi )^{\beta _0 + \beta _1 Z_{t - 1}} \phi ^{Z_t} \right] ,\\{} & {} \displaystyle L_8 \left( \beta _0, \beta _1, \phi \right) = \prod _{t = 2000}^{2021} \left[ {Z_t + \exp \left( \beta _0 + \beta _1 Z_{t - 1}\right) - 1 \atopwithdelims ()Z_t} (1 - \phi )^{\exp \left( \beta _0 + \beta _1 Z_{t - 1}\right) } \phi ^{Z_t} \right] ,\\{} & {} \displaystyle L_9 \left( \beta _0, \beta _1, \beta _2 \right) = \prod _{t = 2000}^{2021} \frac{\left( \beta _0 + \beta _1 Z_{t - 1} + \beta _2 Z_{t - 2}\right) ^{Z_t} \exp \left( -\beta _0 + \beta _1 Z_{t - 1} + \beta _2 Z_{t - 2} \right) }{Z_t!},\\{} & {} \displaystyle L_{10} \left( \beta _0, \beta _1, \beta _2 \right) = \prod _{t = 2000}^{2021} \frac{\exp \left[ Z_t \left( \beta _0 + \beta _1 Z_{t - 1} + \beta _2 Z_{t - 2}\right) \right] \exp \left[ -\exp \left( \beta _0 + \beta _1 Z_{t - 1} + \beta _2 Z_{t - 2}\right) \right] }{Z_t!},\\{} & {} \displaystyle L_{11} \left( \beta _0, \beta _1, \beta _2, \phi \right) = \prod _{t = 2000}^{2021} \left[ {Z_t + \beta _0 + \beta _1 Z_{t - 1} + \beta _2 Z_{t - 2} - 1 \atopwithdelims ()Z_t} (1 - \phi )^{\beta _0 + \beta _1 Z_{t - 1} + \beta _2 Z_{t - 2}} \phi ^{Z_t} \right] ,\\{} & {} \displaystyle L_{12} \left( \beta _0, \beta _1, \beta _2, \phi \right) = \prod _{t = 2000}^{2021} \left[ {Z_t + \exp \left( \beta _0 + \beta _1 Z_{t - 1} + \beta _2 Z_{t - 2}\right) - 1 \atopwithdelims ()Z_t} (1 - \phi )^{\exp \left( \beta _0 + \beta _1 Z_{t - 1} + \beta _2 Z_{t - 2}\right) } \phi ^{Z_t} \right] ,\\{} & {} \displaystyle L_{13} \left( \beta _0, \beta _1 \right) = \prod _{t = 2000}^{2021} \frac{\left( \beta _0 + \beta _1 t\right) ^{Z_t} \exp \left( -\beta _0 - \beta _1 t \right) }{Z_t!},\\{} & {} \displaystyle L_{14} \left( \beta _0, \beta _1 \right) = \prod _{t = 2000}^{2021} \frac{\exp \left[ Z_t \left( \beta _0 + \beta _1 t \right) \right] \exp \left[ -\exp \left( \beta _0 + \beta _1 t \right) \right] }{Z_t!},\\{} & {} \displaystyle L_{15} \left( \beta _0, \beta _1, \phi \right) = \prod _{t = 2000}^{2021} \left[ {Z_t + \beta _0 + \beta _1 t - 1 \atopwithdelims ()Z_t} (1 - \phi )^{\beta _0 + \beta _1 t} \phi ^{Z_t} \right] , \end{aligned}$$

$$\begin{aligned}{} & {} \displaystyle L_{16} \left( \beta _0, \beta _1, \phi \right) = \prod _{t = 2000}^{2021} \left[ {Z_t + \exp \left( \beta _0 + \beta _1 t \right) - 1 \atopwithdelims ()Z_t} (1 - \phi )^{\exp \left( \beta _0 + \beta _1 t \right) } \phi ^{Z_t} \right] ,\\{} & {} \displaystyle L_{17} \left( \beta _0, \beta _1, \beta _2 \right) = \prod _{t = 2000}^{2021} \frac{\left( \beta _0 + \beta _1 t + \beta _2 t^2 \right) ^{Z_t} \exp \left( -\beta _0 - \beta _1 t - \beta _2 t^2 \right) }{Z_t!},\\{} & {} \displaystyle L_{18} \left( \beta _0, \beta _1, \beta _2 \right) = \prod _{t = 2000}^{2021} \frac{\exp \left[ Z_t \left( \beta _0 + \beta _1 t + \beta _2 t^2 \right) \right] \exp \left[ -\exp \left( \beta _0 + \beta _1 t + \beta _2 t^2 \right) \right] }{Z_t!},\\{} & {} \displaystyle L_{19} \left( \beta _0, \beta _1, \beta _2, \phi \right) = \prod _{t = 2000}^{2021} \left[ {Z_t + \beta _0 + \beta _1 t + \beta _2 t^2 - 1 \atopwithdelims ()Z_t} (1 - \phi )^{\beta _0 + \beta _1 t + \beta _2 t^2} \phi ^{Z_t} \right] , \end{aligned}$$

and

$$\begin{aligned} \displaystyle L_{20} \left( \beta _0, \beta _1, \beta _2, \phi \right) = \prod _{t = 2000}^{2021} \left[ {Z_t + \exp \left( \beta _0 + \beta _1 t + \beta _2 t^2 \right) - 1 \atopwithdelims ()Z_t} (1 - \phi )^{\exp \left( \beta _0 + \beta _1 t + \beta _2 t^2 \right) } \phi ^{Z_t} \right] , \end{aligned}$$

respectively, with respect to $\beta _0$, $\beta _1$, $\beta _2$ and $\phi$. We shall denote their maximum likelihood estimates by $\widehat{\beta _0}$, $\widehat{\beta _1}$, $\widehat{\beta _2}$ and ${\widehat{\phi }}$, respectively. The likelihood functions were maximized using the command tsglm in the R package tscount (Liboschik et al.^17^; R Development Core Team^18^).

For each of the fitted models, we computed the Akaike Information Criterion (AIC) due to Akaike^19^, Bayesian Information Criterion (BIC) due to Schwarz^20^ and associated *p*-values of the Kolmogorov–Smirnov test (Kolmogorov^8^; Smirnov^21^) obtained by re-sampling. The values of AIC were computed as

$$\begin{aligned}{} & {} \displaystyle \textrm{AIC} = 2 - 2 \log L_1 \left( \widehat{\beta _0} \right) ,\\{} & {} \displaystyle \textrm{AIC} = 2 - 2 \log L_2 \left( \widehat{\beta _0} \right) ,\\{} & {} \displaystyle \textrm{AIC} = 4 - 2 \log L_3 \left( \widehat{\beta _0}, {\widehat{\phi }} \right) ,\\{} & {} \displaystyle \textrm{AIC} = 4 - 2 \log L_4 \left( \widehat{\beta _0}, {\widehat{\phi }} \right) ,\\{} & {} \displaystyle \textrm{AIC} = 4 - 2 \log L_5 \left( \widehat{\beta _0}, \widehat{\beta _1} \right) ,\\{} & {} \displaystyle \textrm{AIC} = 4 - 2 \log L_6 \left( \widehat{\beta _0}, \widehat{\beta _1} \right) ,\\{} & {} \displaystyle \textrm{AIC} = 6 - 2 \log L_7 \left( \widehat{\beta _0}, \widehat{\beta _1}, {\widehat{\phi }} \right) ,\\{} & {} \displaystyle \textrm{AIC} = 6 - 2 \log L_8 \left( \widehat{\beta _0}, \widehat{\beta _1}, {\widehat{\phi }} \right) ,\\{} & {} \displaystyle \textrm{AIC} = 6 - 2 \log L_9 \left( \widehat{\beta _0}, \widehat{\beta _1}, \widehat{\beta _2} \right) ,\\{} & {} \displaystyle \textrm{AIC} = 6 - 2 \log L_{10} \left( \widehat{\beta _0}, \widehat{\beta _1}, \widehat{\beta _2} \right) ,\\{} & {} \displaystyle \textrm{AIC} = 8 - 2 \log L_{11} \left( \widehat{\beta _0}, \widehat{\beta _1}, \widehat{\beta _2}, {\widehat{\phi }} \right) ,\\{} & {} \displaystyle \textrm{AIC} = 8 - 2 \log L_{12} \left( \widehat{\beta _0}, \widehat{\beta _1}, \widehat{\beta _2}, {\widehat{\phi }} \right) ,\\{} & {} \displaystyle \textrm{AIC} = 4 - 2 \log L_{13} \left( \widehat{\beta _0}, \widehat{\beta _1} \right) ,\\{} & {} \displaystyle \textrm{AIC} = 4 - 2 \log L_{14} \left( \widehat{\beta _0}, \widehat{\beta _1} \right) ,\\{} & {} \displaystyle \textrm{AIC} = 6 - 2 \log L_{15} \left( \widehat{\beta _0}, \widehat{\beta _1}, {\widehat{\phi }} \right) ,\\{} & {} \displaystyle \textrm{AIC} = 6 - 2 \log L_{16} \left( \widehat{\beta _0}, \widehat{\beta _1}, {\widehat{\phi }} \right) ,\\{} & {} \displaystyle \textrm{AIC} = 6 - 2 \log L_{17} \left( \widehat{\beta _0}, \widehat{\beta _1}, \widehat{\beta _2} \right) ,\\{} & {} \displaystyle \textrm{AIC} = 6 - 2 \log L_{18} \left( \widehat{\beta _0}, \widehat{\beta _1}, \widehat{\beta _2} \right) ,\\{} & {} \displaystyle \textrm{AIC} = 8 - 2 \log L_{19} \left( \widehat{\beta _0}, \widehat{\beta _1}, \widehat{\beta _2}, {\widehat{\phi }} \right) , \end{aligned}$$

and

$$\begin{aligned} \displaystyle \textrm{AIC} = 8 - 2 \log L_{20} \left( \widehat{\beta _0}, \widehat{\beta _1}, \widehat{\beta _2}, {\widehat{\phi }} \right) . \end{aligned}$$

The values of BIC were computed as

$$\begin{aligned}{} & {} \displaystyle \textrm{BIC} = \log (22) - 2 \log L_1 \left( \widehat{\beta _0} \right) ,\\{} & {} \displaystyle \textrm{BIC} = \log (22) - 2 \log L_2 \left( \widehat{\beta _0} \right) ,\\{} & {} \displaystyle \textrm{BIC} = 2 \log (22) - 2 \log L_3 \left( \widehat{\beta _0}, {\widehat{\phi }} \right) ,\\{} & {} \displaystyle \textrm{BIC} = 2 \log (22) - 2 \log L_4 \left( \widehat{\beta _0}, {\widehat{\phi }} \right) ,\\{} & {} \displaystyle \textrm{BIC} = 2 \log (22) - 2 \log L_5 \left( \widehat{\beta _0}, \widehat{\beta _1} \right) ,\\{} & {} \displaystyle \textrm{BIC} = 2 \log (22) - 2 \log L_6 \left( \widehat{\beta _0}, \widehat{\beta _1} \right) ,\\{} & {} \displaystyle \textrm{BIC} = 3 \log (22) - 2 \log L_7 \left( \widehat{\beta _0}, \widehat{\beta _1}, {\widehat{\phi }} \right) ,\\{} & {} \displaystyle \textrm{BIC} = 3 \log (22) - 2 \log L_8 \left( \widehat{\beta _0}, \widehat{\beta _1}, {\widehat{\phi }} \right) ,\\{} & {} \displaystyle \textrm{BIC} = 3 \log (22) - 2 \log L_9 \left( \widehat{\beta _0}, \widehat{\beta _1}, \widehat{\beta _2} \right) ,\\{} & {} \displaystyle \textrm{BIC} = 3 \log (22) - 2 \log L_{10} \left( \widehat{\beta _0}, \widehat{\beta _1}, \widehat{\beta _2} \right) ,\\{} & {} \displaystyle \textrm{BIC} = 4 \log (22) - 2 \log L_{11} \left( \widehat{\beta _0}, \widehat{\beta _1}, \widehat{\beta _2}, {\widehat{\phi }} \right) ,\\{} & {} \displaystyle \textrm{BIC} = 4 \log (22) - 2 \log L_{12} \left( \widehat{\beta _0}, \widehat{\beta _1}, \widehat{\beta _2}, {\widehat{\phi }} \right) ,\\{} & {} \displaystyle \textrm{BIC} = 2 \log (22) - 2 \log L_{13} \left( \widehat{\beta _0}, \widehat{\beta _1} \right) ,\\{} & {} \displaystyle \textrm{BIC} = 2 \log (22) - 2 \log L_{14} \left( \widehat{\beta _0}, \widehat{\beta _1} \right) ,\\{} & {} \displaystyle \textrm{BIC} = 3 \log (22) - 2 \log L_{15} \left( \widehat{\beta _0}, \widehat{\beta _1}, {\widehat{\phi }} \right) ,\\{} & {} \displaystyle \textrm{BIC} = 3 \log (22) - 2 \log L_{16} \left( \widehat{\beta _0}, \widehat{\beta _1}, {\widehat{\phi }} \right) ,\\{} & {} \displaystyle \textrm{BIC} = 3 \log (22) - 2 \log L_{17} \left( \widehat{\beta _0}, \widehat{\beta _1}, \widehat{\beta _2} \right) ,\\{} & {} \displaystyle \textrm{BIC} = 3 \log (22) - 2 \log L_{18} \left( \widehat{\beta _0}, \widehat{\beta _1}, \widehat{\beta _2} \right) ,\\{} & {} \displaystyle \textrm{BIC} = 4 \log (22) - 2 \log L_{19} \left( \widehat{\beta _0}, \widehat{\beta _1}, \widehat{\beta _2}, {\widehat{\phi }} \right) , \end{aligned}$$

and

$$\begin{aligned} \displaystyle \textrm{BIC} = 4 \log (22) - 2 \log L_{20} \left( \widehat{\beta _0}, \widehat{\beta _1}, \widehat{\beta _2}, {\widehat{\phi }} \right) . \end{aligned}$$

The best fitting model for each country was determined by values of the AIC, BIC and *p*-values of the Kolmogorov–Smirnov test. The parameter estimates, 95% confidence intervals and values of the AIC, BIC and *p*-values of the Kolmogorov–Smirnov test are given in Table 1 when the best fitted model was the identity Poisson model. The parameter estimates, 95% confidence intervals and values of the AIC, BIC and *p*-values of the Kolmogorov–Smirnov test are given in Table 2 when the best fitted model was the log Poisson model. The parameter estimates, 95% confidence intervals and values of the AIC, BIC and *p*-values of the Kolmogorov-Smirnov test are given in Table 3 when the best fitted model was the log Poisson model regressed linearly versus year. The parameter estimates, 95 percent confidence intervals and values of the AIC, BIC and *p*-values of the Kolmogorov–Smirnov test are given in Table 4 when the best fitted model was the log Poisson model regressed on the previous observation. The parameter estimates, 95 percent confidence intervals and values of the AIC, BIC and *p*-values of the Kolmogorov–Smirnov test are given in Table 5 when the best fitted model was the identity Poisson model regressed on the previous observation. The parameter estimates, 95% confidence intervals and values of the AIC, BIC and *p*-values of the Kolmogorov–Smirnov test are given in Table 6 when the best fitted model was the log negative binomial model regressed on the two previous observations. The parameter estimates, 95 percent confidence intervals and values of the AIC, BIC and *p*-values of the Kolmogorov–Smirnov test are given in Table 7 when the best fitted model was the log negative binomial model regressed linearly versus year. The parameter estimates, 95 percent confidence intervals and values of the AIC, BIC and *p*-values of the Kolmogorov–Smirnov test are given in Table 8 when the best fitted model was the log negative binomial model regressed on the previous observation.

## Results and discussion

Models based on the negative binomial distribution gave the better fit for Sao Tome and Principe, Eritrea, Seychelles, Tanzania, Cabo Verde, Djibouti, Eswatini, Kenya, Lesotho, Malawi, Namibia, South Africa and Zimbabwe. Models based on Poisson distribution gave the better fit for Chad, Comoros, the Democratic Republic of Congo, Mauritius, Morocco, Nigeria, the Central African Republic, Benin, Botswana, Burkina Faso, Burundi, Cameroon, Cote d’Ivoire, Egypt, Equatorial Guinea, Ethiopia, Ghana, Guinea, Liberia, Madagascar, Mali, Mauritania, Niger, Senegal, Sudan, the Gambia, Uganda, Zambia, Gabon, Guinea-Bissau, Mozambique, the Republic of Congo, Rwanda, Sierra Leone, Somalia, Tunisia, Angola, Algeria and Togo.

In the following countries, the newly reported cases of TB exhibits a decreasing trend over 2000 to 2021: Benin, Botswana, Burkina Faso, Burundi, Cameroon, Cote d’Ivoire, Egypt, Ethiopia, Ghana, Guinea, Madagascar, Mali, Mauritania, Niger, Senegal, Sudan, The Gambia, Uganda, Zambia, Eritrea, Seychelles and Tanzania. The decreasing trend appears sharpest for Botswana and shallowest for the Gambia. Equatorial Guinea and Liberia exhibit positive trends in the newly reported cases of TB over 2000 to 2021. Equatorial Guinea has the sharper positive trend.

The number of new cases immediately preceding has a positive impact on the rate of new cases for Gabon, Guinea-Bissau, Mozambique, the Republic of Congo, Rwanda, Sierra Leone, Somalia, Tunisia, Angola, Algeria, Togo, Cabo Verde, Djibouti, Eswatini, Kenya, Lesotho, Malawi, Namibia, South Africa, Zimbabwe, and Sao Tome and Principe. The second to the previous number of new cases has a negative impact on the rate of new cases for Sao Tome and Principe.

**Table 1:** Parameter estimates, 95% confidence intervals and values of the AIC, BIC and *p*-values of the Kolmogorov–Smirnov test when the best fitted model was the identity Poisson model.

Country	Parameter estimate of $\beta _0$ (95% CI)	$\log L$	AIC	BIC	*p*-value
Chad	146.909 (141.844, 151.974)	− 76.190	154.381	155.472	0.149
Comoros	35.273 (32.791, 37.754)	− 60.105	122.209	123.300	0.060
Democratic Republic of Congo	324.955 (317.422, 332.487)	− 84.140	170.281	171.372	0.110
Mauritius	12.682 (11.194, 14.170)	− 49.641	101.283	102.374	0.138
Morocco	101.045 (96.845, 105.246)	− 74.560	151.120	152.211	0.138
Nigeria	219.000 (212.816, 225.184)	− 79.505	161.010	162.101	0.148

**Table 2:** Parameter estimates, 95% confidence intervals and values of the AIC, BIC and *p*-values of the Kolmogorov–Smirnov test when the best fitted model was the log Poisson model.

Country	Parameter estimate of $\beta _0$ (95% CI)	$\log L$	AIC	BIC	*p*-value
Central African Republic	6.292 (6.274, 6.310)	− 89.427	180.855	181.946	0.199

**Table 3:** Parameter estimates, 95% confidence intervals and values of the AIC, BIC and *p*-values of the Kolmogorov–Smirnov test when the best fitted model was the log Poisson model regressed linearly versus year.

Country	Parameter estimates of $\beta _0$ and $\beta _1$ (95% CIs)	$\log L$	AIC	BIC	*p*-value
Benin	47.801 (31.538, 64.064), − 0.022 (− 0.030, − 0.014)	− 66.630	137.260	139.442	0.084
Botswana	143.131 (137.068, 149.195), − 0.068 (− 0.071, − 0.065)	− 97.634	199.268	201.450	0.140
Burkina Faso	46.300 (28.737, 63.863), − 0.021 (− 0.030, − 0.012)	− 64.790	133.581	135.763	0.111
Burundi	109.490 (98.739, 120.241), − 0.052 (− 0.057, − 0.047)	− 83.585	171.170	173.353	0.172
Cameroon	72.154 (63.721, 80.588), − 0.033 (− 0.037, − 0.029)	− 87.499	178.997	181.180	0.100
Cote d’Ivoire	111.773 (102.428, 121.117), − 0.053 (− 0.058, − 0.048)	− 83.156	170.313	172.495	0.193
Egypt	85.652 (53.581, 117.723), − 0.041 (− 0.057, − 0.025)	− 51.760	107.521	109.703	0.089
Equatorial Guinea	− 44.262 (− 53.061, − 35.463), 0.025 (0.020, 0.029)	− 87.930	179.860	182.042	0.062
Ethiopia	121.330 (112.838, 129.822), − 0.058 (− 0.062, − 0.053)	− 86.799	177.599	179.781	0.113
Ghana	50.449 (40.493, 60.406), − 0.023 (− 0.027, − 0.018)	− 77.766	159.532	161.714	0.061
Guinea	33.884 (24.324, 43.443), − 0.014 (− 0.019, − 0.009)	− 79.772	163.545	165.727	0.066
Liberia	− 18.786 (− 26.623, − 10.950), 0.012 (0.008, 0.016)	− 85.254	174.508	176.690	0.199
Madagascar	27.848 (19.443, 36.253), − 0.011 (− 0.015, − 0.007)	− 83.266	170.531	172.713	0.196
Mali	45.085 (28.294, 61.876), − 0.020 (− 0.029, − 0.012)	− 65.721	135.443	137.625	0.127
Mauritania	114.511 (103.100, 125.921), − 0.055 (− 0.060, − 0.049)	− 75.488	154.977	157.159	0.195
Niger	89.171 (76.821, 101.522), − 0.042 (− 0.048, − 0.036)	− 74.063	152.127	154.309	0.184
Senegal	34.286 (22.741, 45.830), − 0.015 (− 0.020, − 0.009)	− 73.962	151.925	154.107	0.186
Sudan	90.294 (77.353, 103.234), − 0.043 (− 0.049, − 0.036)	− 80.590	165.180	167.362	0.200
The Gambia	26.140 (16.172, 36.108), − 0.010 (− 0.015, − 0.005)	− 78.322	160.643	162.825	0.102
Uganda	35.881 (26.919, 44.844), − 0.015 (− 0.020, − 0.011)	− 83.249	170.499	172.681	0.178
Zambia	93.021 (86.958, 99.083), − 0.043 (− 0.046, − 0.040)	− 88.494	180.987	183.169	0.091

**Table 4:** Parameter estimates, 95% confidence intervals and values of the AIC, BIC and *p*-values of the Kolmogorov–Smirnov test when the best fitted model was the log Poisson model regressed on the previous observation.

Country	Parameter estimates of $\beta _0$ and $\beta _1$ (95% CIs)	$\log L$	AIC	BIC	*p*-value
Gabon	0.435 (− 2.084, 2.955), 0.930 (0.531, 1.329)	− 91.972	187.945	190.127	0.077
Guinea-Bissau	1.594 (− 1.354, 4.542), 0.728 (0.226, 1.230)	− 88.395	180.789	182.971	0.053
Mozambique	0.267 (− 4.512, 5.047), 0.954 (0.139, 1.769)	− 84.822	173.643	175.825	0.088
Republic of Congo	0.741 (− 1.904, 3.386), 0.875 (0.432, 1.318)	− 88.383	180.767	182.949	0.065
Rwanda	0.706 (− 0.242, 1.655), 0.838 (0.623, 1.052)	− 76.316	156.633	158.815	0.191
Sierra Leone	0.738 (− 4.670, 6.147), 0.871 (− 0.072, 1.813)	− 83.574	171.149	173.331	0.104
Somalia	1.828 (− 2.042, 5.698), 0.674 (− 0.013, 1.361)	− 83.426	170.853	173.035	0.128
Tunisia	0.233 (− 1.376, 1.841), 0.928 (0.464, 1.393)	− 59.284	122.568	124.750	0.112

**Table 5:** Parameter estimates, 95% confidence intervals and values of the AIC, BIC and *p*-values of the Kolmogorov–Smirnov test when the best fitted model was the identity Poisson model regressed on the previous observation.

Country	Parameter estimates of $\beta _0$ and $\beta _1$ (95% CIs)	$\log L$	AIC	BIC	*p*-value
Angola	58.560 (− 47.257, 164.376), 0.822 (0.525, 1.120)	− 89.681	183.363	185.545	0.147
Algeria	18.496 (− 28.728, 65.721), 0.740 (0.090, 1.390)	− 69.537	143.074	145.256	0.104
Togo	3.289 (− 9.376, 15.954), 0.935 (0.715, 1.155)	− 67.611	139.221	141.404	0.057

**Table 6:** Parameter estimates, 95% confidence intervals and values of the AIC, BIC and *p*-values of the Kolmogorov–Smirnov test when the best fitted model was the log negative binomial model regressed on the two previous observations.

Country	Parameter estimates of $\beta _0$, $\beta _1$ and $\beta _2$ (95% CIs)	$\log L$	AIC	BIC	*p*-value
Sao Tome and Principe	3.088 (1.291, 4.885),	− 94.857	197.713	202.077	0.073
	0.775 (0.377, 1.173),				
	− 0.417 (− 0.812, − 0.023)

**Table 7:** Parameter estimates, 95 percent confidence intervals and values of the AIC, BIC and *p*-values of the Kolmogorov–Smirnov test when the best fitted model was the log negative binomial model regressed linearly versus year.

Country	Parameter estimates of $\beta _0$ and $\beta _1$ (95% CIs)	$\log L$	AIC	BIC	*p*-value
Eritrea	104.044 (79.406, 128.682), − 0.049 (− 0.062, − 0.037)	− 101.492	208.984	212.257	0.133
Seychelles	75.045 (33.052, 117.039), − 0.036 (− 0.057, − 0.015)	− 70.090	146.180	149.453	0.114
Tanzania	92.774 (80.155, 105.394), − 0.043 (− 0.049, − 0.037)	− 108.651	223.302	226.575	0.120

**Table 8:** Parameter estimates, 95% confidence intervals and values of the AIC, BIC and *p*-values of the Kolmogorov–Smirnov test when the best fitted model was the log negative binomial model regressed on the previous observation.

Country	Parameter estimates of $\beta _0$ and $\beta _1$ (95 percent CIs)	$\log L$	AIC	BIC	*p*-value
Cabo Verde	0.778 (− 0.656, 2.212), 0.817 (0.483, 1.151)	− 86.747	179.493	182.766	0.097
Djibouti	0.717 (− 0.591, 2.025), 0.884 (0.670, 1.098)	− 123.274	252.549	255.822	0.115
Eswatini	0.080 (− 0.749, 0.910), 0.988 (0.868, 1.109)	− 134.788	275.576	278.849	0.189
Kenya	0.297 (− 0.457, 1.052), 0.951 (0.828, 1.073)	− 108.623	223.245	226.518	0.086
Lesotho	0.252 (− 0.585, 1.089), 0.962 (0.840, 1.084)	− 121.349	248.699	251.972	0.063
Malawi	0.260 (− 0.641, 1.160), 0.953 (0.795, 1.112)	− 108.229	222.458	225.731	0.134
Namibia	0.574 (− 0.231, 1.379), 0.915 (0.795, 1.034)	− 124.685	255.371	258.644	0.177
South Africa	0.030 (− 0.934, 0.995), 0.995 (0.856, 1.135)	− 126.484	258.967	262.240	0.162
Zimbabwe	0.131 (− 0.371, 0.633), 0.979 (0.896, 1.062)	− 105.543	217.085	220.358	0.192

**Figure 3:**
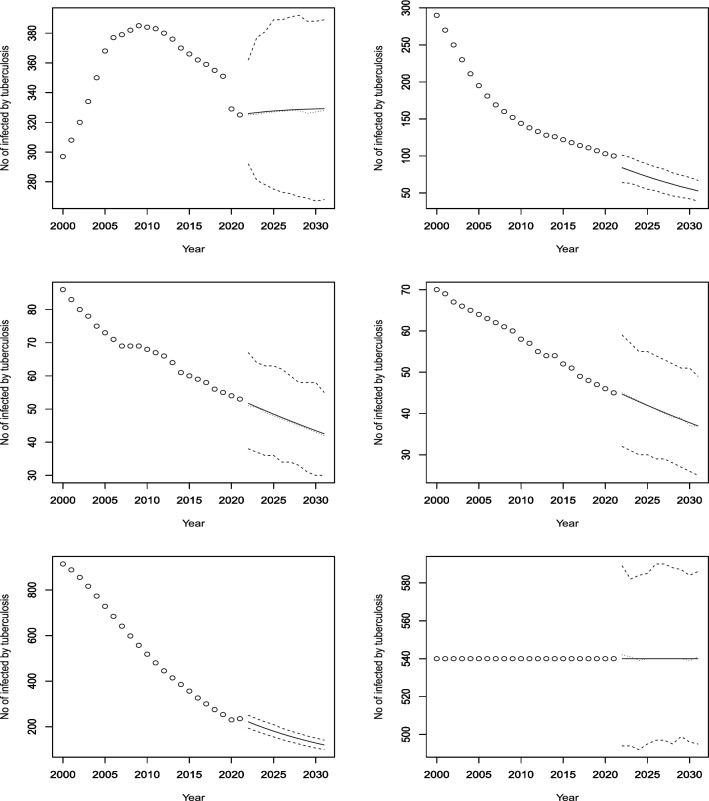
Observed and predicted number infected by TB for Angola (top left), Burundi (top right), Benin (middle left), Burkina Faso (middle right), Botswana (bottom left) and the Central African Republic (bottom right). The predicted number is the solid curve, the median of the predicted number is the curve of dots and the 95 percent confidence interval is the curve of dashes.

**Figure 4:**
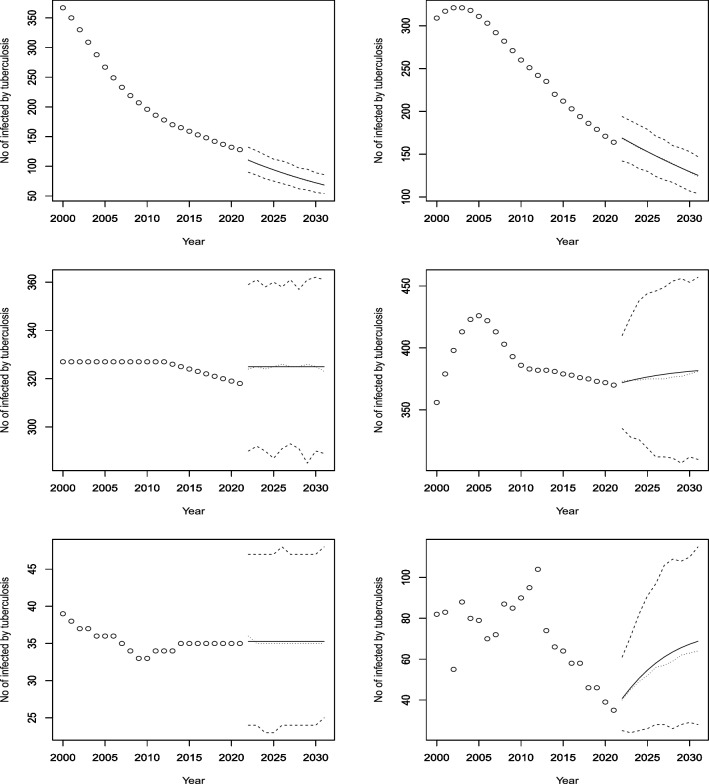
Observed and predicted number infected by TB for Cote d’Ivoire (top left), Cameroon (top right), the Democratic Republic of Congo (middle left), the Republic of Congo (middle right), Comoros (bottom left) and Cabo Verde (bottom right). The predicted number is the solid curve, the median of the predicted number is the curve of dots and the 95% confidence interval is the curve of dashes.

**Figure 5:**
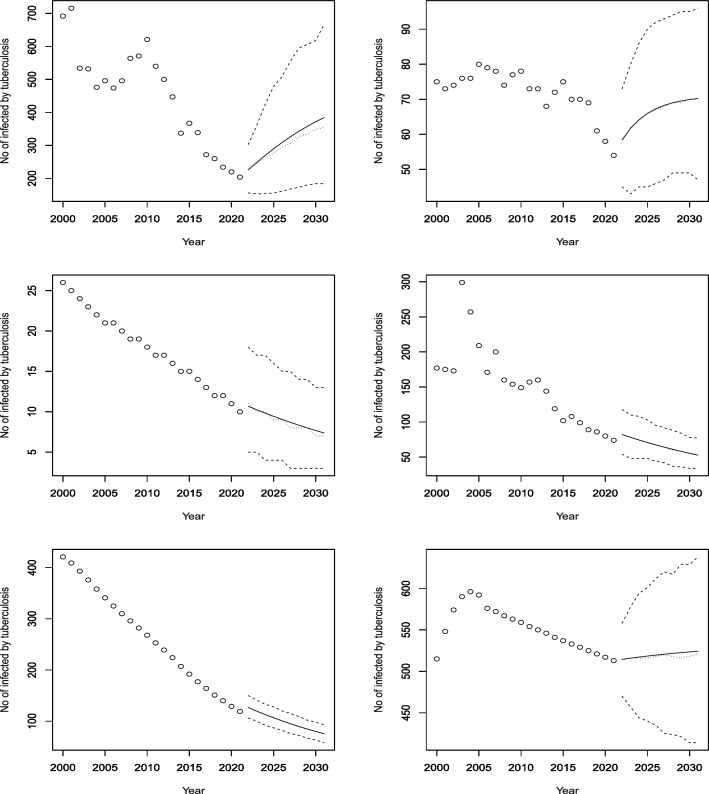
Observed and predicted number infected by TB for Djibouti (top left), Algeria (top right), Egypt (middle left), Eritrea (middle right), Ethiopia (bottom left) and Gabon (bottom right). The predicted number is the solid curve, the median of the predicted number is the curve of dots and the 95% confidence interval is the curve of dashes.

**Figure 6:**
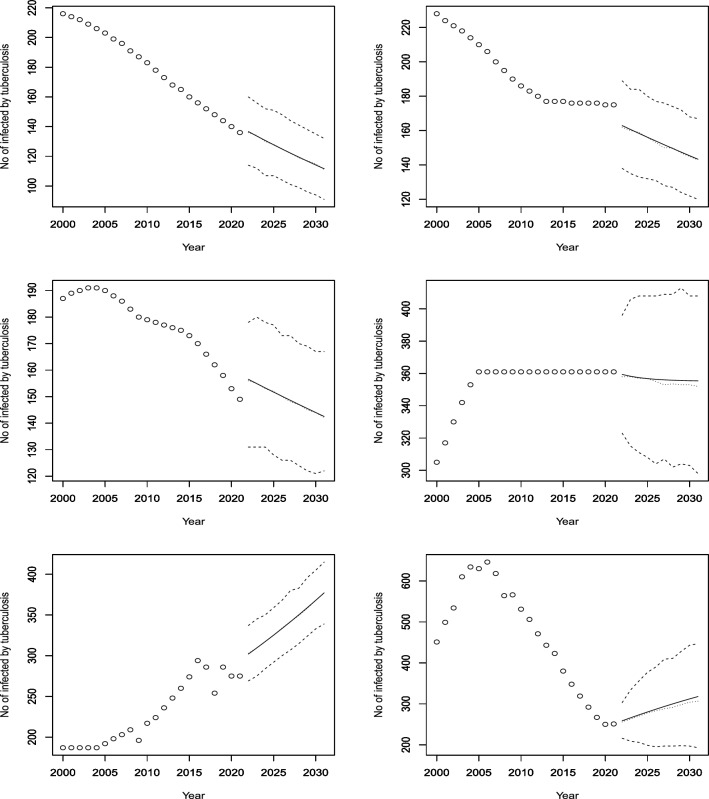
Observed and predicted number infected by TB for Ghana (top left), Guinea (top right), the Gambia (middle left), Guinea-Bissau (middle right), Equatorial Guinea (bottom left) and Kenya (bottom right). The predicted number is the solid curve, the median of the predicted number is the curve of dots and the 95 percent confidence interval is the curve of dashes.

**Figure 7:**
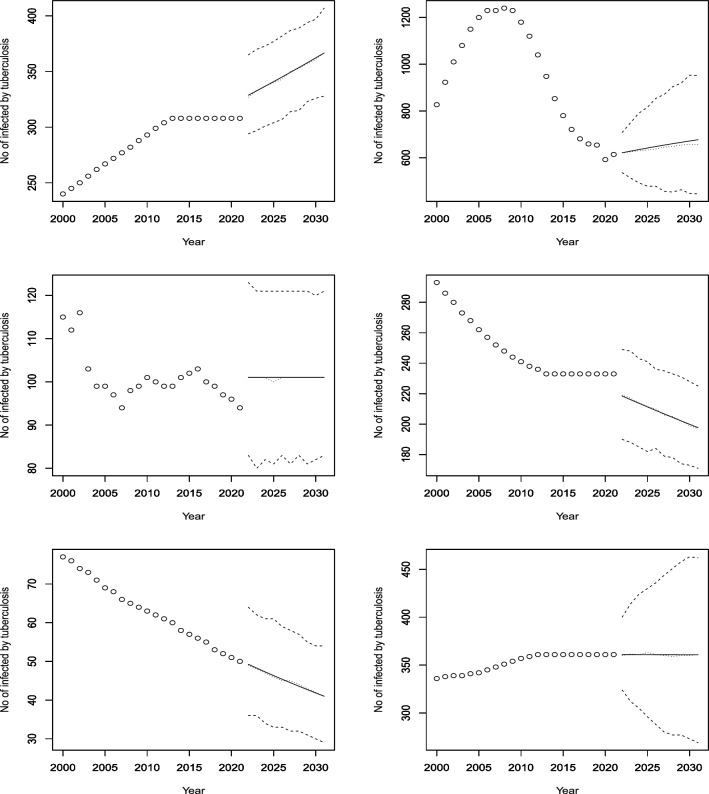
Observed and predicted number infected by TB for Liberia (top left), Lesotho (top right), Morocco (middle left), Madagascar (middle right), Mali (bottom left) and Mozambique (bottom right). The predicted number is the solid curve, the median of the predicted number is the curve of dots and the 95 percent confidence interval is the curve of dashes.

**Figure 8:**
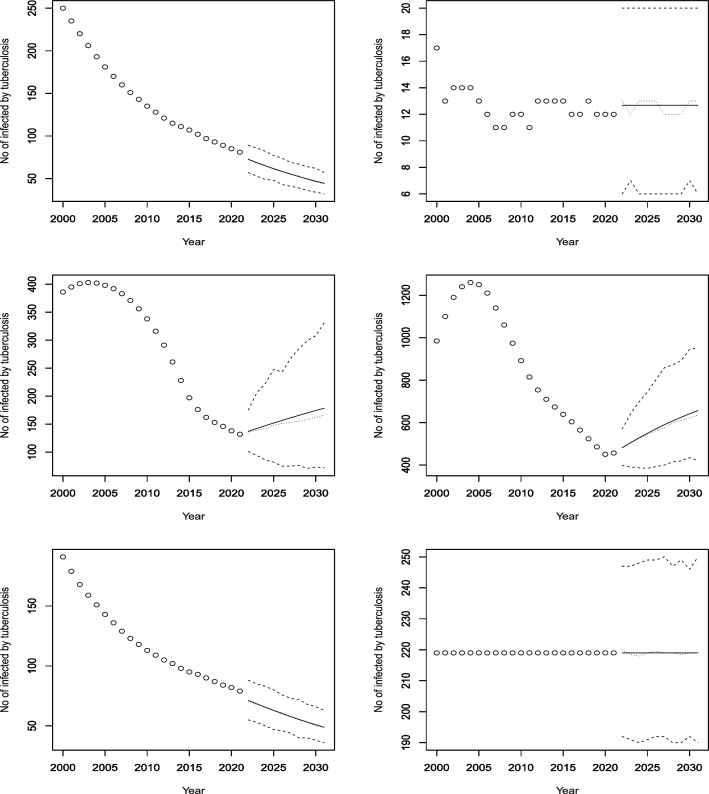
Observed and predicted number infected by TB for Mauritania (top left), Mauritius (top right), Malawi (middle left), Namibia (middle right), Niger (bottom left) and Nigeria (bottom right). The predicted number is the solid curve, the median of the predicted number is the curve of dots and the 95 percent confidence interval is the curve of dashes.

**Figure 9:**
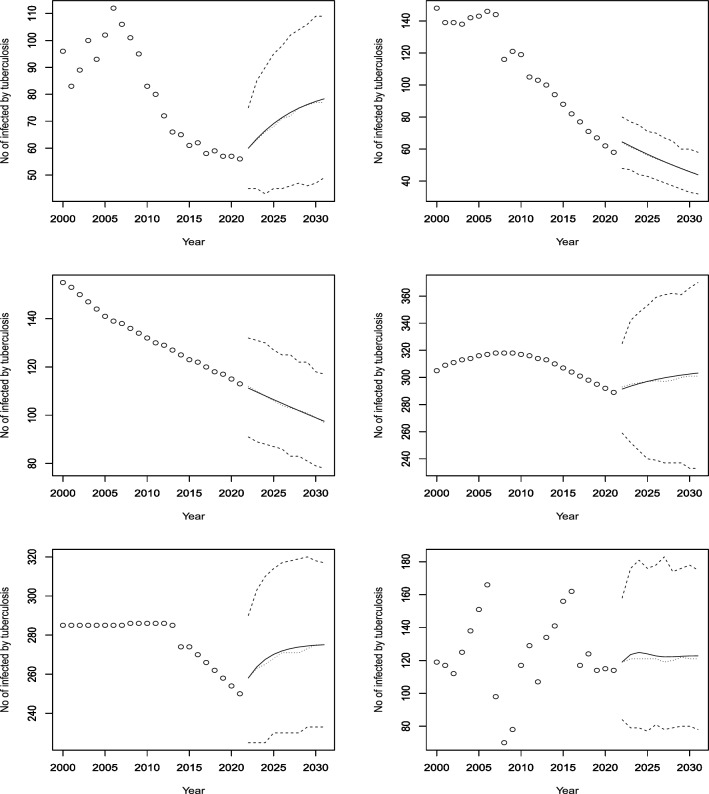
Observed and predicted number infected by TB for Rwanda (top left), Sudan (top right), Senegal (middle left), Sierra Leone (middle right), Somalia (bottom left) and Sao Tome and Principe (bottom right). The predicted number is the solid curve, the median of the predicted number is the curve of dots and the 95 percent confidence interval is the curve of dashes.

**Figure 10:**
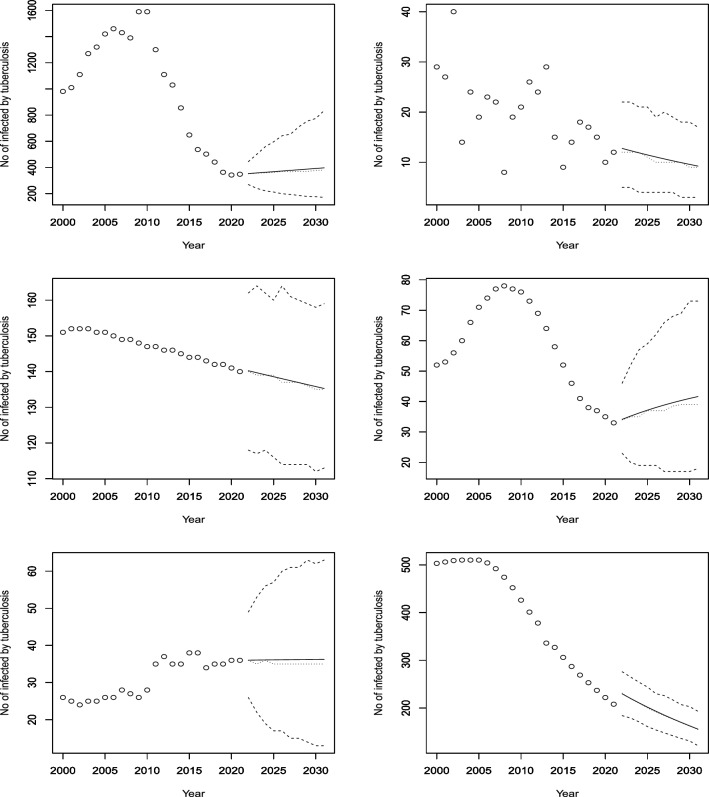
Observed and predicted number infected by TB for Eswatini (top left), Seychelles (top right), Chad (middle left), Togo (middle right), Tunisia (bottom left) and Tanzania (bottom right). The predicted number is the solid curve, the median of the predicted number is the curve of dots and the 95% confidence interval is the curve of dashes.

**Figure 11:**
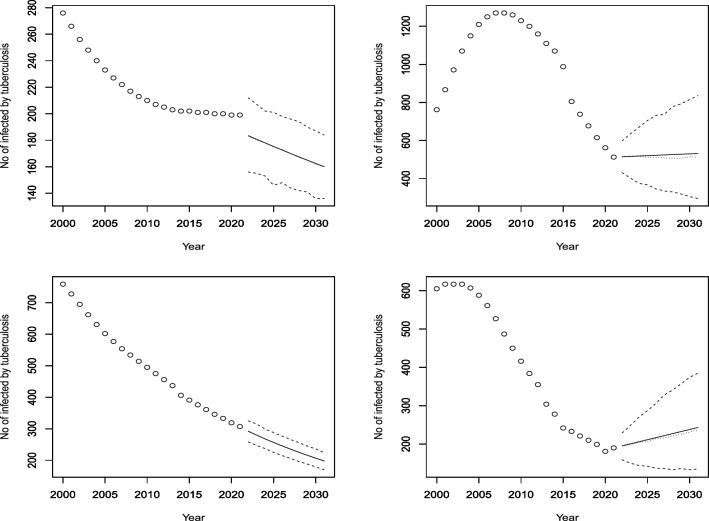
Observed and predicted number infected by TB for Uganda (top left), South Africa (top right), Zambia (bottom left) and Zimbabwe (bottom right). The predicted number is the solid curve, the median of the predicted number is the curve of dots and the 95 percent confidence interval is the curve of dashes.

Figures 3, 4, 5, 6, 7, 8, 9, 10 and 11 plot the predicted new number of cases of TB for the period 2022 to 2031. Also plotted in these figures are the median of the new number of cases and 95% confidence intervals of the new number of cases. For the identity Poisson model, log Poisson model, log Poisson model regressed linearly versus year, identity Poisson model regressed on the previous observation, log Poisson model regressed on the previous observation, log negative binomial model regressed linearly versus year, log negative binomial model regressed on the previous observation and the log negative binomial model regressed on the two previous observations, the predicted new number of cases, say *P*(*t*), was estimated by $\widehat{\beta _0}$, $\exp \left( \widehat{\beta _0} \right)$, $\exp \left( \widehat{\beta _0} + \widehat{\beta _1} t \right)$, $\widehat{\beta _0} + \widehat{\beta _1} Z_{t - 1}$, $\exp \left( \widehat{\beta _0} + \widehat{\beta _1} Z_{t - 1} \right)$, ${\widehat{\phi }} \left( 1 - {\widehat{\phi }} \right) ^{-1} \exp \left( \widehat{\beta _0} + \widehat{\beta _1} t \right)$, ${\widehat{\phi }} \left( 1 - {\widehat{\phi }} \right) ^{-1} \exp \left( \widehat{\beta _0} + \widehat{\beta _1} Z_{t-1} \right)$ and ${\widehat{\phi }} \left( 1 - {\widehat{\phi }} \right) ^{-1} \exp \left( \widehat{\beta _0} + \widehat{\beta _1} Z_{t-1} + \widehat{\beta _2} Z_{t-2} \right)$, respectively, for $t = 2022, 2023, \ldots , 2031$. The corresponding median of the new number of cases, say *M*(*t*), was estimated as the roots of the following equations

$$\begin{aligned}{} & {} \displaystyle \sum _{k = 0}^{M (t)} \frac{{\widehat{\beta _0}}^k \exp \left( -\widehat{\beta _0} \right) }{k!} = 0.5,\\{} & {} \displaystyle \sum _{k = 0}^{M (t)} \frac{\exp \left( k \widehat{\beta _0} \right) \exp \left[ -\exp \left( \widehat{\beta _0} \right) \right] }{k!} = 0.5,\\{} & {} \displaystyle \sum _{k = 0}^{M (t)} \frac{\exp \left[ k \left( \widehat{\beta _0} + \widehat{\beta _1} t \right) \right] \exp \left[ -\exp \left( \widehat{\beta _0} + \widehat{\beta _0} t \right) \right] }{k!} = 0.5,\\{} & {} \displaystyle \sum _{k = 0}^{M(t)} \frac{\left( \widehat{\beta _0} + \widehat{\beta _1} P(t-1) \right) ^k \exp \left( -\widehat{\beta _0} - \widehat{\beta _1} P(t-1) \right) }{k!} = 0.5,\\{} & {} \displaystyle \sum _{k = 0}^{M(t)} \frac{\exp \left[ k \left( \widehat{\beta _0} + \widehat{\beta _1} P(t-1) \right) \right] \exp \left[ -\exp \left( \widehat{\beta _0} + \widehat{\beta _1} P(t-1) \right) \right] }{k!} = 0.5,\\{} & {} \displaystyle \sum _{k = 0}^{M(t)} \left[ {k + \exp \left( \widehat{\beta _0} + \widehat{\beta _1} t \right) - 1 \atopwithdelims ()k} \left( 1 - {\widehat{\phi }} \right) ^{\exp \left( \widehat{\beta _0} + \widehat{\beta _1} t \right) } \left( {\widehat{\phi }} \right) ^k \right] = 0.5,\\{} & {} \displaystyle \sum _{k = 0}^{M(t)} \left[ {k + \exp \left( \widehat{\beta _0} + \widehat{\beta _1} P(t-1) \right) - 1 \atopwithdelims ()k} \left( 1 - {\widehat{\phi }} \right) ^{\exp \left( \widehat{\beta _0} + \widehat{\beta _1} P(t-1) \right) } \left( {\widehat{\phi }} \right) ^k \right] = 0.5, \end{aligned}$$

and

$$\begin{aligned} \displaystyle \sum _{k = 0}^{M(t)} \left[ {k + \exp \left( \widehat{\beta _0} + \widehat{\beta _1} P(t-1) + \widehat{\beta _2} P(t-2) \right) - 1 \atopwithdelims ()k} \left( 1 - {\widehat{\phi }} \right) ^{\exp \left( \widehat{\beta _0} + \widehat{\beta _1} P(t-1) + \widehat{\beta _2} P(t-2) \right) } \left( {\widehat{\phi }} \right) ^k \right] = 0.5, \end{aligned}$$

for $t = 2022, 2023, \ldots , 2031$. The corresponding 95% confidence interval of the new number of cases, say $\left[ L (t), U (t) \right]$, was estimated as the roots of the following equations

$$\begin{aligned}{} & {} \displaystyle \sum _{k = 0}^{L (t), U(t)} \frac{{\widehat{\beta _0}}^k \exp \left( -\widehat{\beta _0} \right) }{k!} = 0.025, 0.975,\\{} & {} \displaystyle \sum _{k = 0}^{L (t), U(t)} \frac{\exp \left( k \widehat{\beta _0} \right) \exp \left[ -\exp \left( \widehat{\beta _0} \right) \right] }{k!} = 0.025, 0.975,\\{} & {} \displaystyle \sum _{k = 0}^{L (t), U(t)} \frac{\exp \left[ k \left( \widehat{\beta _0} + \widehat{\beta _1} t \right) \right] \exp \left[ -\exp \left( \widehat{\beta _0} + \widehat{\beta _0} t \right) \right] }{k!} = 0.025, 0.975,\\{} & {} \displaystyle \sum _{k = 0}^{L (t), U(t)} \frac{\left( \widehat{\beta _0} + \widehat{\beta _1} P(t-1) \right) ^k \exp \left( -\widehat{\beta _0} - \widehat{\beta _1} P(t-1) \right) }{k!} = 0.025, 0.975,\\{} & {} \displaystyle \sum _{k = 0}^{L (t), U(t)} \frac{\exp \left[ k \left( \widehat{\beta _0} + \widehat{\beta _1} P(t-1) \right) \right] \exp \left[ -\exp \left( \widehat{\beta _0} + \widehat{\beta _1} P(t-1) \right) \right] }{k!} = 0.025, 0.975,\\{} & {} \displaystyle \sum _{k = 0}^{L (t), U(t)} \left[ {k + \exp \left( \widehat{\beta _0} + \widehat{\beta _1} t \right) - 1 \atopwithdelims ()k} \left( 1 - {\widehat{\phi }} \right) ^{\exp \left( \widehat{\beta _0} + \widehat{\beta _1} t \right) } \left( {\widehat{\phi }} \right) ^k \right] = 0.025, 0.975,\\{} & {} \displaystyle \sum _{k = 0}^{L (t), U(t)} \left[ {k + \exp \left( \widehat{\beta _0} + \widehat{\beta _1} P(t-1) \right) - 1 \atopwithdelims ()k} \left( 1 - {\widehat{\phi }} \right) ^{\exp \left( \widehat{\beta _0} + \widehat{\beta _1} P(t-1) \right) } \left( {\widehat{\phi }} \right) ^k \right] = 0.025, 0.975, \end{aligned}$$

and

$$\begin{aligned} \displaystyle \sum _{k = 0}^{L (t), U(t)} \left[ {k + \exp \left( \widehat{\beta _0} + \widehat{\beta _1} P(t-1) + \widehat{\beta _2} P(t-2) \right) - 1 \atopwithdelims ()k} \left( 1 - {\widehat{\phi }} \right) ^{\exp \left( \widehat{\beta _0} + \widehat{\beta _1} P(t-1) + \widehat{\beta _2} P(t-2) \right) } \left( {\widehat{\phi }} \right) ^k \right] = 0.025, 0.975, \end{aligned}$$

for $$t = 2022, 2023, \ldots , 2031$$.

According to Fig. 3, four African countries (Burundi, Benin, Burkina Faso and Botswana) have seen a steady decline in their infected populations since the early 2000s. Botswana’s decline has been especially sharp since 2005, and despite a slight rise during the COVID-19 pandemic, it is expected to continue dropping until 2030, though at a slower pace. Burkina Faso and Benin are projected to maintain their historical rates of decline, while Burundi is likely to accelerate its reduction of cases. Angola, on the other hand, had a rapid increase in infections from 2000 to 2010, followed by a decrease until 2020. The model predicts a modest rise in cases for Angola in the next decade. The Central African Republic has shown no significant change in its infection rate for the past 20 years, and the model does not anticipate any changes by 2030.

Figure 4 shows that Cote d’Ivoire and Cameroon are projected to have rising infection rates, which is in line with their historical trends over the past 18 years. The Democratic Republic of Congo and Comoros, however, are expected to have stable infection rates in the future (DRC rates remain constant after a sudden jump). The former had a nearly constant number of cases for the first 11 years, followed by a decade of decline. The latter had a decreasing trend for the first decade, then a non-decreasing trend for the next five years, and then a constant rate. The Republic of Congo, which had been declining for the last 10 years, is forecasted to have a slight increase in cases. Cabo Verde, which had fluctuating infection rates for the first 13 years and then non-increasing rates for the next nine years, is predicted to have an increasing trend until at least 2030.

According to Fig. 5, the model forecasts a decrease in cases for the next decade or so, based on the consistent decline over the years for both Ethiopia and Egypt. Eritrea, which has a less consistent historical trend than Ethiopia and Egypt, is also projected to have a reduction in incidences. On the other hand, Djibouti, Algeria, and Gabon are expected to have an increase in cases, despite having some periods of decline in the past. This is an interesting observation that warrants further investigation.

Figure 6 shows that Ghana has shown a consistent decrease in its infection rates over the years, and the model anticipates a continued decline. Guinea, which had a decrease from 2000 to 2010 and then a stable rate until 2021, is also forecasted to have a decline in cases. Gambia, which had a varying rate of decrease from 2005 to 2021, is expected to have a lower rate of decrease starting from a higher level than the last historical point. Guinea Bissau, which had an increase until 2005 and then a constant rate until 2021, is projected to have a slight dip and then a stable rate. Equatorial Guinea, which had mostly increasing rates with some outliers, is predicted to have a further increase in cases. Kenya, which had a decreasing trend for the last 15 years, is forecasted to reverse its trend and have an increasing rate until 2030.

According to Fig. 7, Liberia had an increasing trend for 13 years until it stabilized at around 308 cases per 100,000 people. The model predicts a further increase with high confidence. Lesotho had a quadratic increase until 2007, followed by a period of stagnation and then a decrease, possibly due to interventions for drug-resistant TB (Satti et al.^22^). The model forecasts a reversal of the decreasing trend after 2022. Morocco had fluctuating rates over the past two decades, but the model expects a rise and then a stabilization at around 100 cases. Mozambique had a constant rate of 361 cases, with wide confidence intervals, and the model does not anticipate any change. Mali and Madagascar are expected to decrease their incidence rates with relatively high confidence.

According to Fig. 8, Mauritania and Niger had decreasing trends from 2000 to 2021, possibly due to the World Health Organization’s End TB strategy launched in 2016 and 2019, respectively (Aw et al.^23^). The model predicts similar rates of decline for both countries. Mauritius had a fluctuating trend, with peaks and troughs every two years. The model forecasts a stable rate, with a constant mean but a piecewise constant median. Malawi and Namibia had similar patterns, with a decrease in cases around 2005 and then a stabilization. The model forecasts increasing trends for both countries from 2022 to 2030. Nigeria had a stable rate from 2000 to 2021, and the model expects no change in the future. The observed and forecasted patterns are consistent for Mauritius and Nigeria. Nigeria is predicted to have a constant incidence of TB at around 220 per 100,000 people.

According to Fig. 9, Rwanda had a fluctuating but generally increasing trend until 2008, when it started to decline. The model predicts significant increases in the future. Sudan and Senegal had decreasing trends and are expected to continue this decline. Sierra Leone and Somalia had decreasing trends in the last 10 years, but are forecasted to have increasing rates in the next decade. Sao Tome and Principe had a cyclical pattern of increases and decreases, and is projected to have a slight increase and then a stabilization until 2030.

According to Fig. 10, Eswatini had an increasing trend for the first 10 years, followed by a 15-year period of decline, except for two years with a significant rise. The model forecasts modest increases from 2022 to 2030. Seychelles had fluctuating rates but is expected to have a decline in rates. Togo had a concave-down curve, with a peak around 2005 and then a decline. The model predicts a rise in cases in the future. Chad had a decreasing trend throughout the dataset, and the model expects a continued decline. Tunisia had a relatively stable rate, and the model does not anticipate any change. Tanzania had a decreasing trend, and the model projects a further decrease until 2030.

According to Fig. 11, Uganda had a stable rate of infection for about 10 years, but the model forecasts a sharp decline from 2022 to 2030. South Africa had a very high rate of TB until 2010, when it started to decline steadily. The model predicts a slightly increasing, stable rate for the next 8 years. Zambia had a rapid decline in infection rates from 2000 to 2021, and the model expects a continued decrease in the future. Zimbabwe had a decreasing trend since 2004, but the model forecasts an increase in cases in the next decade.

Angola (slightly), Algeria, Democratic Republic of Congo, Cameroon, Cabo Verde, Djibouti, Equatorial Guinea, Eswatini, Gabon, Kenya, Malawi, Namibia, Rwanda, Sao Tome and Principe, Sierra Leone, Somalia, Togo, Zimbabwe are expected to experience increases in TB infection rates. TB cases increasing in Equatorial Guinea could be due to lack of important knowledge about TB and bad attitudes of caregivers, making TB one of the major causes of morbidity and mortality in Equatorial Guinea (Vericat-Ferrer et al.^24^). According to Kitimo^25^, a representative of the Intergovernmental Authority on Development Mission to Kenya was quoted as saying that mixed migration flows and lack of government data sharing mechanism had resulted in ineffective anti-TB campaigns in the region. Cross-border movement is viewed as a factor in the TB infection rates in Kenya and a significant barrier to the fight against the disease. It is however important to note that Ethiopia, a neighboring country, is expected to experience a decrease in incidences.

Southern Africa accounts for a third of the world’s countries with highest TB burdens. It is also a hotspot for TB/HIV co-infections with Mozambique, Malawi, Lesotho and Zambia being significant actors (World Bank^26^).

To help mitigate Southern Africa’s complex TB and HIV epidemics and the closely related occupational lung diseases, the World Bank Board of Directors, on 19 June 2020, approved $56 million additional financing from the International Development Association (IDA). This new financing brought the total World Bank financing for the Southern Africa Tuberculosis and Health Systems Support Project (SATBHSSP) to $178 million, covering Lesotho, Malawi, Mozambique and Zambia with the goal of enhancing TB case detection and treatment (World Bank^26^). Our projections, however, predict a rise in TB infections for all these countries except Zambia, where we forecast a steady decrease. This may not necessarily be a bad thing if we find that the injection of funds led to higher detection rates.

In recent years, Southern Africa began turning the tide against TB (World Health Organization^3,5^). Six countries in Southern Africa achieved reductions of 4–10% per year in TB incidence following a peak in the HIV epidemic. These countries are Botswana, Eswatini, Lesotho, Namibia, South Africa and Zimbabwe. As mentioned earlier, models show that Eswatini, Malawi, Namibia, Lethoso, South Africa, and Zimbabwe are predicting increases in TB cases. Mozambique is virtually constant and Botswana and Madagascar are being projected to decrease.

We note that incidences of TB in North Africa seem much less than in other regions. Algeria, Egypt, Morocco, Sudan, and Tunisia had 54, 10, 94, 58, and 36 cases per 100,000 people, respectively. Reasons for such relatively less incidence rates may include the fact that North African countries tend to be more economically developed than other African countries translating into more resources to invest in including TB control and other public health measures. Superior access to health care helps with diagnosis and treatment and lower HIV prevalence—a major risk factor—contributes to lower rates of TB. Our models forecast a slight increase followed by a decrease in Sudan, gradual and consistent decrease in Mauritania, a somewhat sharp increase immediately followed by a period of stagnation in Morocco, a concave increase in Algeria, a small increase followed by years of decreasing incidence rates in Egypt, and virtually no change in Tunisia. Eltayeb et al.^27^ found that for successful TB control in the Middle East and North Africa regions, TB awareness and interventions targeting the elderly and those from lower-in-come settings, particularly directed at gender differences, are essential.

One limitation of our study could be the quality of our data. It is also worth noting that many of the countries have suboptimal case detection rate percentages. This means that the numbers obtained by the World Bank are almost certainly underestimating the true incidence rates. Another important limitation is that the models in some cases seem sensitive to the result of upticks in incidences following the COVID-19 pandemic.

## Conclusions

We have modeled the new cases of TB reported in $2000, 2001, \ldots , 2021$ for fifty two African countries. We used integer time series models due to Fokianos et al.^12^, Fokianos and Tjostheim^13^, Fokianos and Fried^14^ and Christou and Fokianos^15,16^ based on the negative binomial and Poisson distributions.

Based on the best fitted models, we were able to obtain point estimates as well as prediction intervals of incidence rates for years starting in 2022 till 2031. Except in rare instances, the prediction intervals were rather wide. An epidemiological model could have provided less variable conditions (although this would be based on many assumptions made).

Assuming the forecasts are accurate, the model predicts how the infection rates may change or stay the same without interventions. Countries with rising or stable rates should act more forcefully to reduce infections. Countries with falling rates should not relax their efforts, but keep following their current strategies.

The results show that the fear of certain researchers about the attainability of the SDG goals are founded. The forecasting shows that TB incidences will not be reduced by 90% unless decisive action is taken by policy makers and health care practitioners. Tunisia, Niger, Mauritania, and Egypt are the only countries who may have a realistic chance of eliminating TB.

According to Silva et al.^6^, recommendations to combat TB could include: augmenting domestic financial resources, forging mutually beneficial alliances with the private sector, securing more international financial assistance, establishing venues for interaction, emulating efficacious practices at national and regional levels (African Union^28^), and expeditiously developing and adopting novel tools, interventions, and strategies.

To fight TB, governments, organizations, and influential people need to join forces with the same alacrity, political commitment, and resolve displayed during the COVID-19 pandemic. The improved ability to test and sequence genes that was developed to fight COVID-19 can help find TB cases better, a key step in stopping the disease from spreading and helping those who have it survive (World Health Organization^29^).

Finally, a new vaccine would help to drastically reduce the incidence of TB. In countries where TB is common, Bacillle Calmette-Guerin (BCG)—the only licensed TB vaccine in the world—is effective in protecting against TB meningitis and disseminated TB. However, it has not been very effective for teens and adults as well as those with pulmonary TB, particularly in developing countries around the world. A new vaccine is needed and should be efficacious in protecting against developing TB thus reducing spread and also leading to drastic reductions in mortality. According to Davenne and McShane^30^, one idea that may lead to better immunization is the use aerosol vaccines. Such vaccines reproduce the natural route of infection of Mtb, as they directly target alveolar macrophages.

## Data Availability

The data can be obtained from the corresponding author.

## Code availability

The codes can be obtained from the corresponding author.

## References

[1] Centers for Disease Control and Prevention. *Tuberculosis (TB)*. https://www.cdc.gov/tb/topic/basics/default.htm (2023).

[2] Dutta, S. *History of Tuberculosis*. https://www.news-medical.net/health/History-of-Tuberculosis.aspx (2023).

[3] World Health Organization (2022). Global Tuberculosis Report 2022.

[4] Chakaya J, Khan M, Ntoumi F, Aklillu E, Fatima R, Mwaba P, Kapata N, Mfinanga S, Hasnain SE, Katoto PDMC, Bulabula ANH, Sam-Agudu NA, Nachega JB, Tiberi S, McHugh TD, Abubakar I, Zumla A (2021). Global tuberculosis report 2020—Reflections on the Global TB burden, treatment and prevention efforts. Int. J. Infect. Dis..

[5] World Health Organization. *Tuberculosis Deaths Rise for the First Time in More Than a Decade Due to the COVID-19 Pandemic*. https://www.who.int/news/item/14-10-2021-tuberculosis-deaths-rise-for-the-first-time-in-more-than-a-decade-due-to-the-covid-19-pandemic (2021).

[6] Silva S, Arinaminpathy N, Atun R, Goosby E, Reid M (2021). Economic impact of tuberculosis mortality in 120 countries and the cost of not achieving the Sustainable Development Goals tuberculosis targets: A full-income analysis. Lancet Glob. Health.

[7] Ozcaglar C, Shabbeer A, Vandenberg SL, Yener B, Bennett KP (2012). Epidemiological models of *Mycobacterium tuberculosis* complex infections. Math. Biosci..

[8] Kolmogorov A (1933). Sulla determinazione empirica di una legge di distribuzione. Giornale dell’Ist. Ital. degli Attuari.

[9] Liu L, Zhao XQ, Zhou Y (2010). A tuberculosis model with seasonality. Bull. Math. Biol..

[10] Liu Q, Li Z, Ji Y, Martinez L, Zia UH, Javaid A, Lu W, Wang J (2019). Forecasting the seasonality and trend of pulmonary tuberculosis in Jiangsu Province of China using advanced statistical time-series analyses. Infect. Drug Resist..

[11] World Bank. *Tuberculosis Incidence (per 100,000 people)*. https://databank.worldbank.org/metadataglossary/health-nutrition-and-population-statistics/series/SH.TBS.INCD (2023).

[12] Fokianos K, Rahbek A, Tjostheim D (2009). Poisson autoregression. J. Am. Stat. Assoc..

[13] Fokianos K, Tjostheim D (2011). Log-linear Poisson autoregression. J. Multivar. Anal..

[14] Fokianos K, Fried R (2012). Interventions in log-linear Poisson autoregression. Stat. Model..

[15] Christou V, Fokianos K (2014). Quasi-likelihood inference for negative binomial time series models. J. Time Ser. Anal..

[16] Christou V, Fokianos K (2015). Estimation and testing linearity for non-linear mixed Poisson autoregressions. Electron. J. Stat..

[17] Liboschik, T., Fried, R., Fokianos, K. Probst, P. & Rathjens, J. *tscount: Analysis of Count Time Series*. https://CRAN.R-project.org/package=tscount (2020).

[18] R Development Core Team. *R: A Language and Environment for Statistical Computing* (R Foundation for Statistical Computing, 2023).

[19] Akaike H (1974). A new look at the statistical model identification. IEEE Trans. Autom. Control.

[20] Schwarz GE (1978). Estimating the dimension of a model. Ann. Stat..

[21] Smirnov N (1948). Table for estimating the goodness of fit of empirical distributions. Ann. Math. Stat..

[22] Satti H, Seung K, Keshavjee S, Furin J (2008). Extensively drug-resistant tuberculosis, Lesotho. Emerg. Infect. Dis..

[23] Aw B, Ade S, Hinderaker SG, Dlamini N, Takarinda KC, Chiaa K, Feil A, Traore A, Reid T (2017). Childhood tuberculosis in Mauritania, 2010–2015: Diagnosis and outcomes in Nouakchott and the rest of the country. Public Health Action.

[24] Vericat-Ferrer M, Ayala A, Ncogo P, Eyene-Acuresila J, Garcia B, Benito A, Roma-Barja M (2022). Knowledge, attitudes, and stigma: The perceptions of tuberculosis in Equatorial Guinea. Int. J. Environ. Res. Public Health.

[25] Kitimo, A. *Porous Border Blamed for TB Cases in Kenya and Ethiopia. The East African*. https://www.theeastafrican.co.ke/tea/science-health/porous-border-blamed-for-tb-cases-in-kenya-and-ethiopia-3764090 (2022).

[26] World Bank. *Scaling Up Support to Help Combat Tuberculosis and Occupational Lung Diseases in Southern Africa*. https://www.worldbank.org/en/news/press-release/2020/06/19/scaling-up-support-to-help-combat-tuberculosis-and-occupational-lung-diseases-in-southern-africa (2020).

[27] Eltayeb D, Pietersen E, Engel M, Abdullahi L (2020). Factors associated with tuberculosis diagnosis and treatment delays in Middle East and North Africa: A systematic review. East. Mediterr. Health J..

[28] African Union (2016). Africa Health Strategy 2016–2030.

[29] Olle-Goig JE (2010). Tuberculosis in rural Uganda. Afr. Health Sci..

[30] Davenne T, McShane H (2016). Why don’t we have an effective tuberculosis vaccine yet?. Expert Rev. Vaccines.

